# Live-attenuated *Salmonella enterica* serotype Choleraesuis vaccine with regulated delayed *fur* mutation confer protection against *Streptococcus suis* in mice

**DOI:** 10.1186/s12917-020-02340-4

**Published:** 2020-05-07

**Authors:** Yu-an Li, Yunyun Chen, Yuan zhao Du, Weiwei Guo, Dianfeng Chu, Juan Fan, Xiaobo Wang, Matthew Bellefleur, Shifeng Wang, Huoying Shi

**Affiliations:** 1 College of Veterinary Medicine, Yangzhou University, Yangzhou, 225009 Jiangsu People’s Republic of China; 2 Jiangsu Co-innovation Center for the Prevention and Control of Important Animal Infectious Diseases and Zoonoses, Yangzhou, 225009 China; 3 Key Laboratory of Animal Infectious Diseases, Ministry of Agriculture, Yangzhou University, Yangzhou, China; 4 Jiangsu Key Laboratory of Preventive Veterinary Medicine, Yangzhou University, Yangzhou, China; 5 Yebio Bioengineering Co., Ltd of Qingdao, Qingdao, 266114 China; 6 Yangzhou Uni-Bio Pharmaceutical Co., Ltd, Yangzhou, 225000 Jiangsu China; 7 Department of Infectious Diseases and Immunology, College of Veterinary Medicine, University of Florida, Gainesville, FL 32611-0880 USA

**Keywords:** *Salmonella* Choleraesuis, Virulence, Immunogenicity, Fur, Inflammatory

## Abstract

**Background:**

Recombinant *Salmonella enterica* serotype Choleraesuis (*S*. Choleraesuis) vaccine vector could be used to deliver heterologous antigens to prevent and control pig diseases. We have previously shown that a live-attenuated *S*. Choleraesuis vaccine candidate strain rSC0011 (ΔP_crp527_::TT *araC* P_BAD_*crp* Δ*pmi-2426* Δ*relA199*::*araC* P_BAD_*lacI* TT Δ*asdA33*, Δ, deletion, TT, terminator) delivering SaoA, a conserved surface protein in most of *S*. *suis* serotypes, provided excellent protection against *S. suis* challenge, but occasionally lead to morbidity (enteritidis) in vaccinated mice (approximately 1 in every 10 mice). Thus, alternated attenuation method was sought to reduce the reactogenicity of strain rSC0011. Herein, we described another recombinant attenuated *S.* Choleraesuis vector, rSC0012 (ΔP_fur88_:: TT *araC* P_BAD_*fur* Δ*pmi-2426* Δ*relA199*:: *araC* P_BAD_*lacI* TT Δ*asdA33*) with regulated delayed *fur* mutation to avoid inducing disease symptoms while exhibiting a high degree of immunogenicity.

**Results:**

The strain rSC0012 strain with the ΔP_fur88_::TT *araC* P_BAD_*fur* mutation induced less production of inflammatory cytokines than strain rSC0011 with the ΔP_crp527_::TT *araC* P_BAD_*crp* mutation in mice. When delivering the same pS-SaoA plasmid, the intraperitoneal LD_50_ of rSC0012 was 18.2 times higher than that of rSC0011 in 3-week-old BALB/C mice. rSC0012 with either pS-SaoA or pYA3493 was cleared from spleen and liver tissues 7 days earlier than rSC0011 with same vectors after oral inoculation. The strain rSC0012 synthesizing SaoA induced high titers of anti-SaoA antibodies in both systemic (IgG in serum) and mucosal (IgA in vaginal washes) sites, as well as increased level of IL-4, the facilitator of Th2-type T cell immune response in mice. The recombinant vaccine rSC0012(pS-SaoA) conferred high percentage of protection against *S. suis* or *S*. Choleraesuis challenge in BALB/C mice.

**Conclusions:**

The live-attenuated *Salmonella enterica* serotype Choleraesuis vaccine rSC0012(pS-SaoA) with regulated delayed *fur* mutation provides a foundation for the development of a safe and effective vaccine against *S*. Choleraesuis and *S. suis*.

## Background

*Streptococcus suis* is a pandemic pathogen responsible for a wide range of invasive diseases such as pneumonia, meningitis and bacteraemia in both humans and pigs [1, 2]. *S. suis* type 2 (SS2) is the most frequently and virulent isolated from both humans and pigs among all serotypes reported to date [1, 3]. The surface-anchored protein (Sao) is a highly conserved membrane-anchored protein and proved to be a immunogenic vaccine candidate [4]. However, Sao formulated with Emulsigen-Plus® provides only partial protection to mice against SS2 infection [3]. In our previous study, a recombinant attenuated *Salmonella enterica* serotype Choleraesuis vaccine strain rSC0016 carrying *saoA* gene, provided full protection to mice against SS2 challenge [5]. From the above, an effective delivery system such as live *Salmonella enterica* serotype Choleraesuis play a crucial role to the effectiveness of Sao.

The use of intracellular *Salmonella enterica* as a vehicle to deliver heterologous protective antigens against pathogens is an attractive strategy. Curtiss et al. developed the RDAS (Regulated Delayed Attenuated Strategies), which enable live *salmonella* vaccine effectively colonize lymphoid tissues during the invasion stage because of its wild-type aggressiveness and then be full attenuated by silencing the virulence factor, while stimulate both strong cellular and humoral immunity in the immunized mice [6]. Several ways were used to implement this strategy (RDAS). One way is the reverse synthesis of lipopolysaccharide O-antigen by *pmi* mutation [7]. Another way is to replace the upstream regulatory and promoter sequences of virulence genes with a tightly regulated *araC* P_BAD_ activator–promoter [8]. This strategy has been successfully used for *S*. Typhimurium and *S*. Typhi [6–8]. With this strategy, we construct a regulated delayed *S.* Choleraesuis vaccine strain rSC0011 with ΔP_crp527_::TT *araC* P_BAD_*crp* and *pmi* mutations [9]. rSC0011 delivering *S. suis* antigens were effective to induce protective immunity against SS2 in mice, but it occasionally caused enteritidis.

We sought to improve our *S*. Choleraesuis candidate vector vaccine by using alternative mutation or introducing new mutation to decreasing its potential to induce enteritidis and enhance immunogenicity. Fur is an important regulatory protein in *Salmonella*. In the presence of iron, Fur acts as a repressor of iron-controlled genes and mounts an adaptive acid tolerance response [8]. Synthesis of Fur in a vaccine strain during growth confers acid tolerance and maintains iron homeostasis. A decrease of Fur synthesis in *Salmonella* leads to acid sensitivity and iron acquisition [9]. Curtiss et al. reported that a *S.* Typhimurium strain with an arabinose regulated delayed *fur* mutation is highly immunogenic [6]. In these consideration, an arabinose regulated delayed *fur* mutation (ΔP_fur88_:: TT *araC* P_BAD_*fur*) was introduced into a *S*. Choleraesuis vaccine strain with multiple preexist mutations (Δ*pmi-2426* Δ*relA199::araC* P_BAD_*lacI* TT Δ*asdA33*) to generate strain rSC0012. A plasmid pS-SaoA [5], encoding *saoA* from *SS*2, was transformed into this strain. We evaluated the virulence, immunogenicity and protection against challenge with virulent *SS*2 or *S*. Choleraesuis C78–3.

## Results

### Construction and characterization of the *S.* Choleraesuis vaccine strain rSC0012

**Fig. 1:**
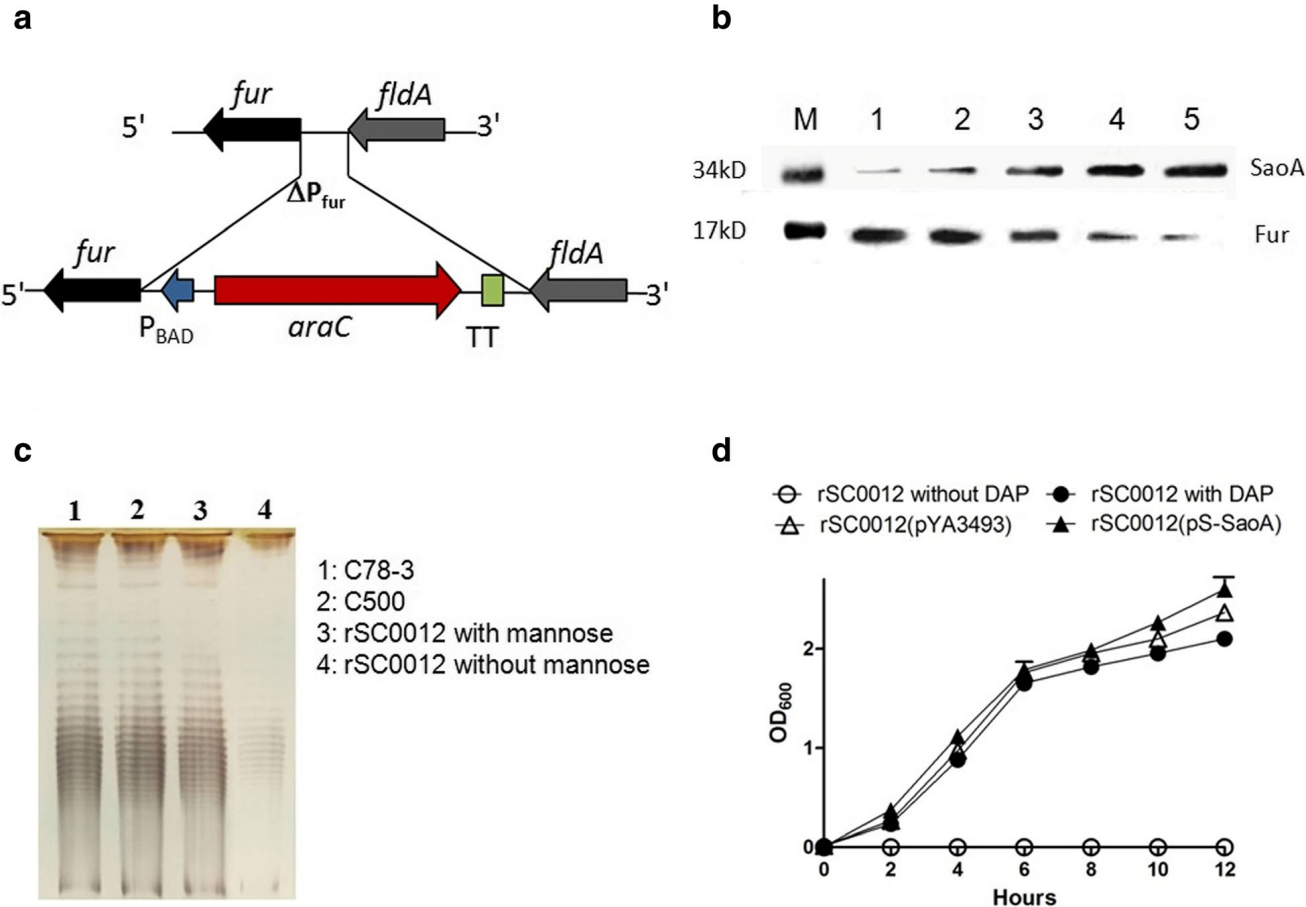
Diagram of chromosomal mutation and phenotypes of the *S*. Choleraesuis vaccine strain rSC0012. (**a**) Schematic map of ΔP_fur_::TT *araC* P_BAD_*fur* deletion - insertion mutation; (**b**) Regulated decreased synthesis of Fur (ΔP_fur_::TT *araC* P_BAD_*fur*) and regulated delayed synthesis of SaoA in rSC0012(pS-SaoA) with the Δ*relA::araC* P_BAD_*lacI* TT mutation. The rSC0012(pS-SaoA) strain was grown in NB with arabinose (Lane 1) when OD_*600*_ reach 0.8 and then diluted at a 1:10 ratio into fresh NB without arabinose. The process continued for 4 times (Lane 2- 5); each lane was loaded around 4.6 × 10^7^ CFU cells. Synthesis of Fur and SaoA were detected by western blot using corresponding antiserum. M: protein marker; (**c**) LPS profile of Δ*pmi* mutation in rSC0012 in NB grown with or without 0.2% mannose. Lanes: 1, wild type C78-3; 2, C500; 3, rSC0012 with mannose; 4, rSC0012 without mannose; (**d**) Growth curves of rSC0012 with and without DAP, rSC0012(pYA3493) or rSC0012(pS-SaoA) in LB were measured with a spectrophotometer (OD_600_) at the indicated time intervals

Fur is a ferric uptake regulator that is involved not only in iron metabolism, uptake, and transport, but also invasion and survival of *S.* Typhimurium in the hosts [10–12]. The absence of Fur attenuates *S.* Typhimurium [6, 13]. To improve the safety and increase the immunogenicity of *S.* Choleraesuis vector, a new strain, rSC0012, was generated with an arabinose regulated *fur*, ΔP_fur88_:: TT *araC* P_BAD_*fur* (Fig. 1a).

The phenotypes of the mutations ΔP^fur88^::TT *araC* P^BAD^*fur* and Δ*relA*::*araC* P^BAD^*lacI* TT were confirmed by western blot analysis (Fig. 1b). The level of Fur synthesis decreased with arabinose dilution (Fig. 1b). The presence of mutation Δ*relA*::*araC* P^BAD^*lacI* TT in rSC0012(pS-SaoA) were confirmed by the increased synthesis of SaoA (Fig. 1b) due to the derepression of P_trc_ promoter on plasmid in rSC0012, which resulted from reduced LacI production whose production was controlled by arabinose. The *pmi* gene encodes 6-phosphomannose isomerase that interconverts fructose-6-phosphate and mannose-6-phosphatein in *Salmonella* [7]. Because mannose is required for O-antigen synthesis, the Δ*pmi* mutation enables the strain rSC0012 to display a smooth LPS pattern in nutrient broth in the presence of mannose and a rough pattern in the absence of mannose (Fig. 1c). The Δ*asdA* mutation enables the strain rSC0012 to have an obligate requirement for DAP [14], which can be complemented with a vector harboring the *asd* gene then eliminates the need for antibiotic resistance genes for plasmid maintenance [15]. The growth rates of rSC0012 with DAP, rSC0012(pS-SaoA), and rSC0012(pYA3493) were similar (Fig. 1d). rSC0012 could grow only with DAP (Fig. 1d).

### Antigen synthesis and plasmid stability in *S. Choleraesuis* rSC0012

Stable maintenance of plasmids and the production of heterologous antigens are critical to ensure efficacy of recombinant live vaccines. The SaoA protein is a highly conserved surface protective antigen among *S. suis* serotypes [2, 5]. Using live attenuated *S.* Choleraesuis vector delivering SaoA antigen from *S. suis* will allow to develop a bivalent vaccine against both *S*. Choleraesuis and *S*. *suis*. The stabilities of pS-SaoA and pYA3493 in rSC0012 were evaluated by continuous culturing for 50 generations. The stabilities of both Asd^+^ plasmids, pS-SaoA and pYA3493, were 100% in rSC0012 (data not shown). All rSC0012 colonies examined (100 clones/generation) by endonuclease digestion possessed the Asd + plasmid pS-SaoA or pYA3493. The 34-kDa SaoA protein was detected in cells obtained from both the first and 50th generations of rSC0012(pS-SaoA) (data not shown), indicating the stability of plasmid and stable synthesis of SaoA.

### Distribution of secreted SaoA in *S.* Choleraesuis rSC0012

**Fig. 2:**
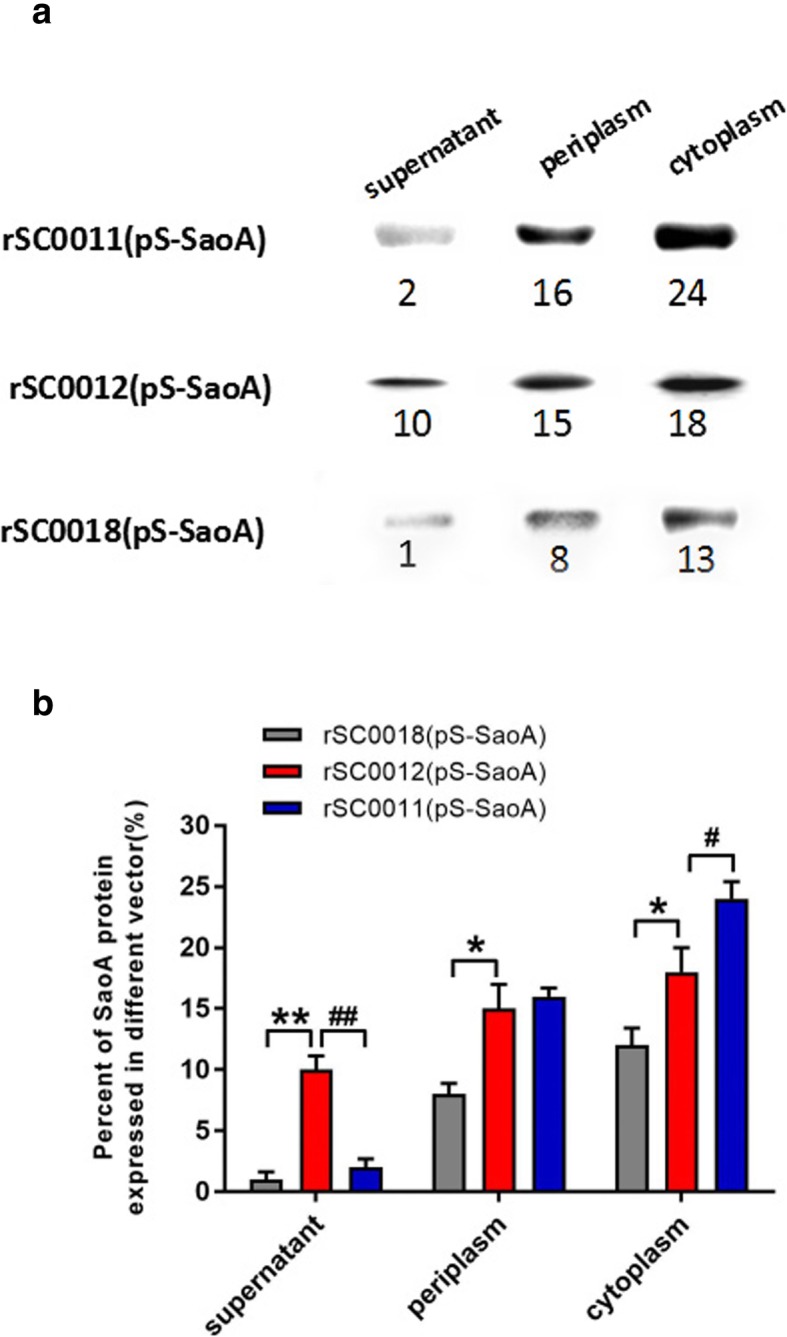
Synthesis of SaoA in *Salmonella* Choleraesuis vector rSC0011, rSC0012 and rSC0018. (**a**) Subcellular fractions of SaoA in rSC0011(pS-SaoA), rSC0012(pS-SaoA), and rSC0018(pS-SaoA) from cells grown in NB detected by western blot. The number showed relative densitometry in one of the three representative experiments. (**b**) Densitometry analysis of the SaoA protein in rSC0011(pSSaoA), rSC0012(pS-SaoA), and rSC0018(pS-SaoA) by using Image J software (Image J2 PMID 26153368). The assay was repeated 3 times. *P < 0.05, **P < 0.01. #,*P* <0.05, ##,*P* < 0.01, for rSC0012(pS-SaoA) compared to rSC0011(pS-SaoA)

The production levels of SaoA in various subcellular fractions from pS-SaoA-carrying strains, rSC0011, rSC0012, and rSC0018, were determined (Fig. 2a-b). rSC0018 is a derivative of C500 [5, 16], a licensed live *S.* Choleraesuis vaccine attenuated by chemical methods in China, with an *asdA* mutation. The results showed that the level of the SaoA protein produced in the cytoplasm by strain rSC0012(pS-SaoA) was significantly lower than strain rSC0011(pS-SaoA) (Fig. 2b; #, *P* < 0.05), but significantly higher in supernatant when compared with rSC0011(pS-SaoA) and rSC0018(pS-SaoA) (Fig. 2b; **, *P* < 0.01). The SaoA protein produced in the periplasm fraction of rSC0012(pS-SaoA) was significantly higher than strain rSC0018(pS-SaoA) (Fig. 2a-b).

### Lower virulence of *S.* Choleraesuis rSC0012 in vivo

To evaluate the virulence of strains with mutations ΔP_fur88_:: TT *araC* P_BAD_*fur* or ΔP_crp527_::TT *araC* P_BAD_*crp*, the LD_50_ values of rSC0012 and rSC0011 were tested in 3-week-old BALB/c mice, which represents young mice. rSC0018 was used as an attenuation control. All strains carried the expression plasmid pS-SaoA. The results revealed that the LD_50_s of rSC0011(pS-SaoA), rSC0012(pS-SaoA), and rSC0018(pS-SaoA) were at least 10^9^ CFU by oral inoculation; whereas, the LD_50_ of wild-type C78–3 was 9.5 × 10^2^ CFU (Table 1). Following intraperitoneal infection, the LD_50_ of rSC0012(pS-SaoA) was 38.89-fold higher than that of rSC0011(pS-SaoA) and 3.2-fold higher than that of rSC0018(pS-SaoA) in mice. These results indicated that the virulence of rSC0012(pS-SaoA) harboring ΔP_fur88_:: TT *araC* P_BAD_*fur* was significantly lower than that of rSC0011(pS-SaoA) harboringΔP_crp527_::TT *araC* P_BAD_*crp* by intraperitoneal route in young mice.

**Table 1:** Virulence of rSC0012(pS-SaoA) in 3-week-old BALB/c mice

Strain	Description	LD_50_ (CFU)	
		Oral	i.p
C78–3	Wild type	9.5 × 10^2^	< 10
rSC0018(pS-SaoA)	Δ*asdA33* in a live attenuated *S.* Choleraesuis vaccine strain C500	> 2.8 × 10^9^	6.6 × 10^6^ **, ##
rSC0012(pS-SaoA)	ΔP_fur88_::TT *araC* P_BAD_*fur* Δ*pmi-2426* Δ*relA199*::*araC* P_BAD_*lacI* TT Δ*asdA33* in C78–3	> 5.8 × 10^9**^	2.1 × 10^7^ **, ##
rSC0011(pS-SaoA)	ΔP_crp527_::TT *araC* P_BAD_*crp* Δ*pmi-2426* Δ*relA199*::*araC* P_BAD_*lacI* TT Δ*asdA33* in C78–3	> 1.0 × 10^9**^	5.4 × 10^5^ **

^**^, *P* < 0.01, compared with C78–3; ^##^, *P* < 0.01, compared with rSC0011 (pS-SaoA)

### Tissue distribution of *S.* Choleraesuis strains in BALB/c mice

**Fig. 3:**
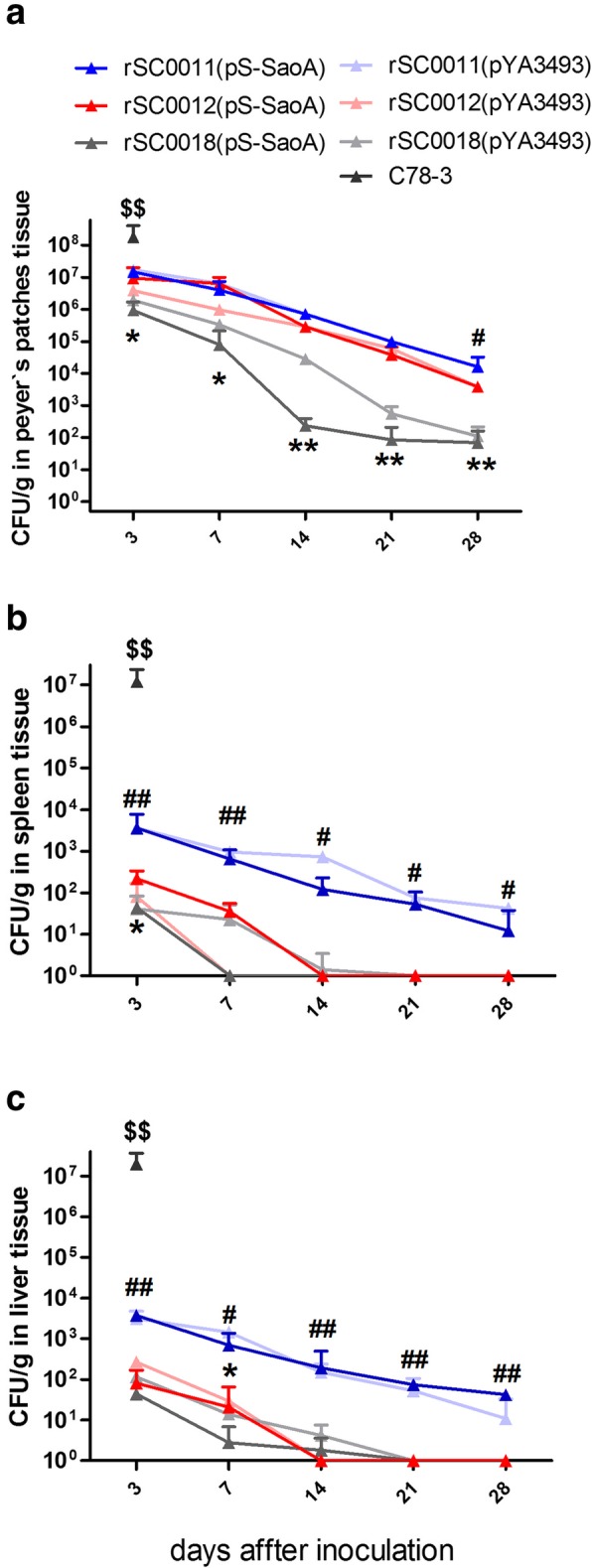
Colonization of *Salmonella* Choleraesuis rSC0012 in BALB/c mice at diferent time points. Mice were orally inoculated with 1.0 ± 0.3 × 10^9^ CFU of the indicated strains. The numbers of bacteria loads in the Peyer’s patches (**a**), spleen (**b**) and liver (**c**) of mice at 3 d, 7 d, 14 d, 21 d and 28 d after inoculation were plotted. Bars represent the arithmetic mean ± standard deviations from two separate experiments each with 5 mice per group. *, *P* < 0.05; **, *P* < 0.01, for rSC0018 compared to rSC0012 or to rSC0011 with either pYA3493 or pS-SaoA; #, *P* < 0.05, ##, *P* < 0.01, for rSC0011 compared to rSC0012 or to rSC0011 with either pYA3493 or pS-SaoA; $$, *P* < 0.01, for C78-3 compared to rSC0011, rSC0012 and rSC0018 with either pYA3493 or pS-SaoA, as indicated. The data were collected from two independent experiments

Fur and Crp are important regulatory proteins in *Salmonella*. Inactivation of the *fur* and *crp* genes, attenuates the organism [6, 17]. To quantitatively the colonization of the *S.* Choleraesuis strains containing regulated delayed *fur* or *crp* mutations, 3-week-old BALB/c mice were orally inoculated with rSC0011(pYA3493), rSC0012(pYA3493), rSC0018(pYA3493), rSC0011(pS-SaoA), rSC0012(pS-SaoA), rSC0018(pS-SaoA), or wild-type C78–3 (Fig. 3a–f). The mice inoculated with wild-type *S.* Choleraesuis C78–3 died 3–5 days after inoculation, whereas the mice infected orally with vaccine strains rSC0011, rSC0012, rSC0018 containing either plasmid pS-SaoA or pYA3493 survived. The bacteria titers of wild-type strain C78–3 in Peyer’s patches, spleen, and liver were significantly higher than those of vaccine strains rSC0011, rSC0012, rSC0018 containing either plasmid pS-SaoA or pYA3493 at 3 days after inoculation (Fig. 3a–c). The titers of bacteria in Peyer’s patches were similar for strains rSC0012(pYA3493), rSC0012(pS-SaoA), rSC0011(pYA3493), and rSC0011(pS-SaoA) at 3–21 days post-inoculation (Fig. 3a). At 3d, 7 d, 14 d, 21 d and 28 d, the numbers of rSC0012(pS-SaoA) were significantly higher than those of attenuated vaccine strains rSC0018(pS-SaoA). Same for two control vector, the numbers of rSC0012(pYA3493) were significantly higher than those of attenuated vaccine strains rSC0018(p YA3493) and there was no significant difference with same strain with expression or control vector. Respectively (*P* < 0.01; Fig. 3a). These results indicating that the colonization abilities of strains rSC0012(pS-SaoA) and rSC0012(pYA3493) in Peyer’s patches were higher than that of rSC0018 with either pYA3493 or pS-SaoA.

In spleen, the titers of rSC0012(pYA3493) and rSC0012(pS-SaoA) were similar to those of vaccine strains rSC0018(pYA3493) and rSC0018(pS-SaoA) at 7, 14, 21, 28 days after inoculation (Fig. 3b). The titers of strains rSC0011(pYA3493) and rSC0011(pS-SaoA) were significantly higher than those of rSC0012(pYA3493), rSC0012(pS-SaoA), rSC0018(pYA3493), and rSC0018(pS-SaoA), at 3, 7, 14, and 21 days, respectively (Fig. 3b).

In liver, the titers of rSC0012(pYA3493) and rSC0012(pS-SaoA) were similar to those of vaccine strains rSC0018(pYA3493) and rSC0018(pS-SaoA) at 3, 14, 21, 28 days after inoculation (Fig. 3c). At 3d, 7 d, 14 d, 21 d and 28 d post-inoculation, the titers of rSC0012(pS-SaoA) were significantly lower than those of rSC0011(pS-SaoA), same for two control vector, the numbers of rSC0012(pYA3493) were significantly lower than those of strains rSC0011(pYA3493).respectively(*P* < 0.01; Fig. 3c), whereas the strain rSC0012(pYA3493) was the fastest to be cleared in liver (Fig. 3c). These results indicated that the ΔP_fur88_:: TT *araC* P_BAD_*fur* mutation impaired the colonization in the liver of *S*. Choleraesuis vaccine strains. In a summary, the ΔP_fur88_:: TT *araC* P_BAD_*fur* mutation reduced the colonization ability of *S.* Choleraesuis vaccine strains in mice spleen and liver but not in the Peyer’s patches.

### Antibody responses in mice immunized with *S.* Choleraesuis strains

**Fig. 4:**
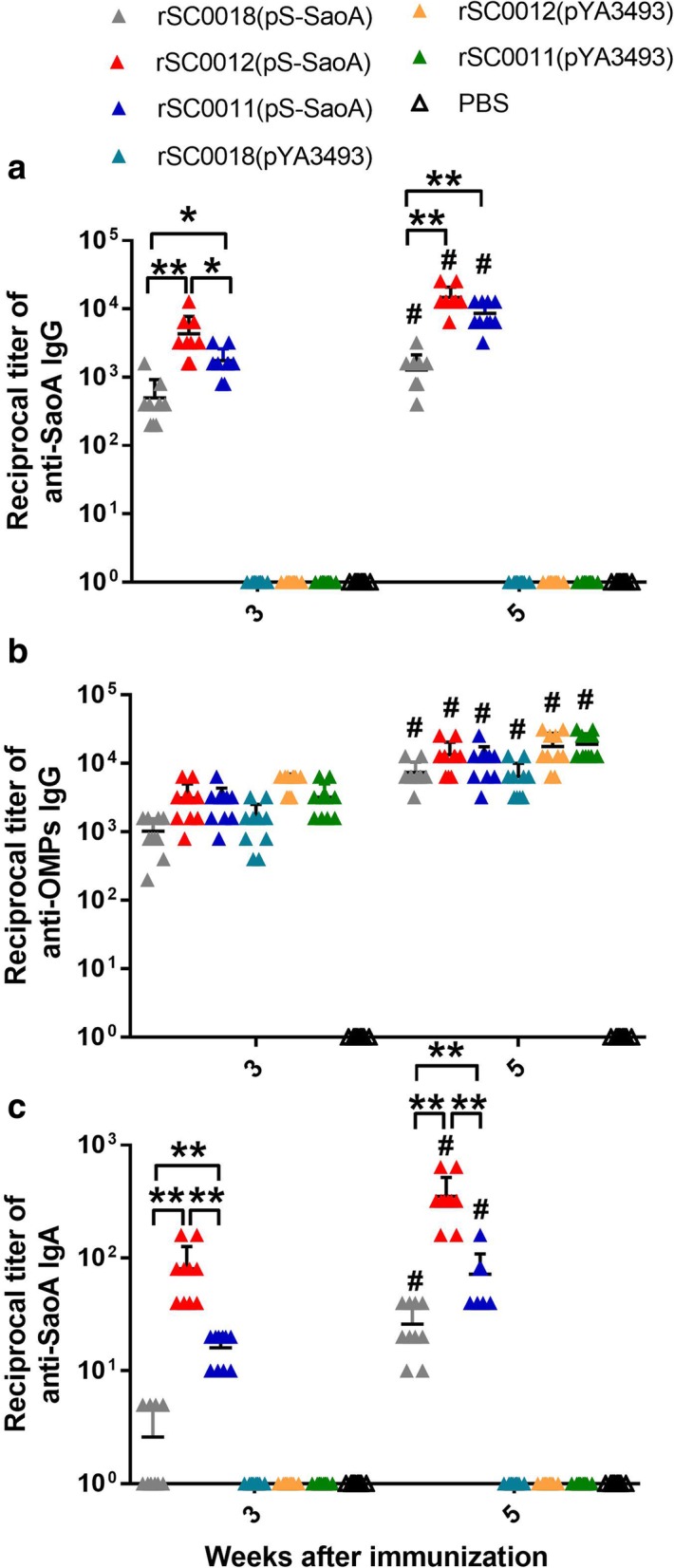
Antibody responses in ten mice. Serum IgG responses to SaoA (**a**), *S*. Choleraesuis OMPs (**b**) and vaginal wash IgA responses to SaoA (**c**) were measured by ELISA at weeks 3 and 5. Each triangle represents one mouse. Error bars represent variation between mice. Significant differences were indicated. *, *P* < 0.05; ** *P* < 0.01, for *Salmonella* carrying pS-SaoA compared to each other; #, *P* < 0.05; ##, *P* < 0.01, for the titers of antibody at 3 weeks after immunization were compared to those at 5 weeks after immunization. No immune responses were detected to antigen tested in mice immunized with PBS or in pre-immune sera from vaccinated mice (reciprocal titer <1:50). The assay was performed in duplicate and repeated at least 3 times

All of the mice immunized with strains containing pS-SaoA developed anti-SaoA antibodies (Fig. 4a). rSC0012(pS-SaoA) induced significantly higher anti-SaoA IgG titer than did rSC0011(pS-SaoA) 3 weeks after the immunization in 3-week-old mice (Fig. 4a; *, *P* < 0.05). Although the anti-SaoA IgG titer of rSC0012(pS-SaoA) were slightly higher than that of rSC0011(pS-SaoA) 5 weeks after immunization, they were not significantly different (Fig. 4a). Compared to mice immunized with rSC0018(pS-SaoA), higher serum IgG titers against SaoA were detected in mice immunized with both rSC0011(pS-SaoA) and rSC0012(pS-SaoA) at 3 weeks and 5 weeks postimmunization (Fig. 4a, *, *P* < 0.05, **, *P* < 0.01). After boosting, higher titers of anti-SaoA IgG were observed in mice immunized with all three strains containing pS-SaoA (Fig. 4a, #, *P* < 0.05). All three attenuated recombinant Salmonella strains induced significant OMP titers after the first immunization in mice (Fig. 4b). A significant boosting of serum antibody responses to OMPs was observed after the second immunization, (Fig. 4b; #, P < 0.05).

Mucosal IgA anti-SaoA responses were detected at week 3 in mice immunized with all three attenuated strains containing pS-SaoA. rSC0018(pS-SaoA) induced lower titers of anti-SaoA IgA than rSC0012(pS-SaoA) or rSC0011(pS-SaoA) did in mice (Fig. 4c, *, P < 0.05, **, *P* < 0.01). Anti-SaoA IgA levels detected in the rSC0012(pS-SaoA) immunized group were significantly higher than those induced in the rSC0011(pS-SaoA) immunized group at 3 and 5 weeks after the immunization (Fig. 4c, *, P < 0.05, **, P < 0.01). These results indicated that strain rSC0012(pS-SaoA) harboring ΔP_fur88_:: TT *araC* P_BAD_*fur* elicited a stronger immune response than strain rSC0011(pS-SaoA) harboring ΔP_crp527_::TT *araC* P_BAD_*crp* did, especially elicit a stronger mucosal immune response.

### IFN-γ or IL-4 production induced by *S.* Choleraesuis strains

**Fig. 5:**
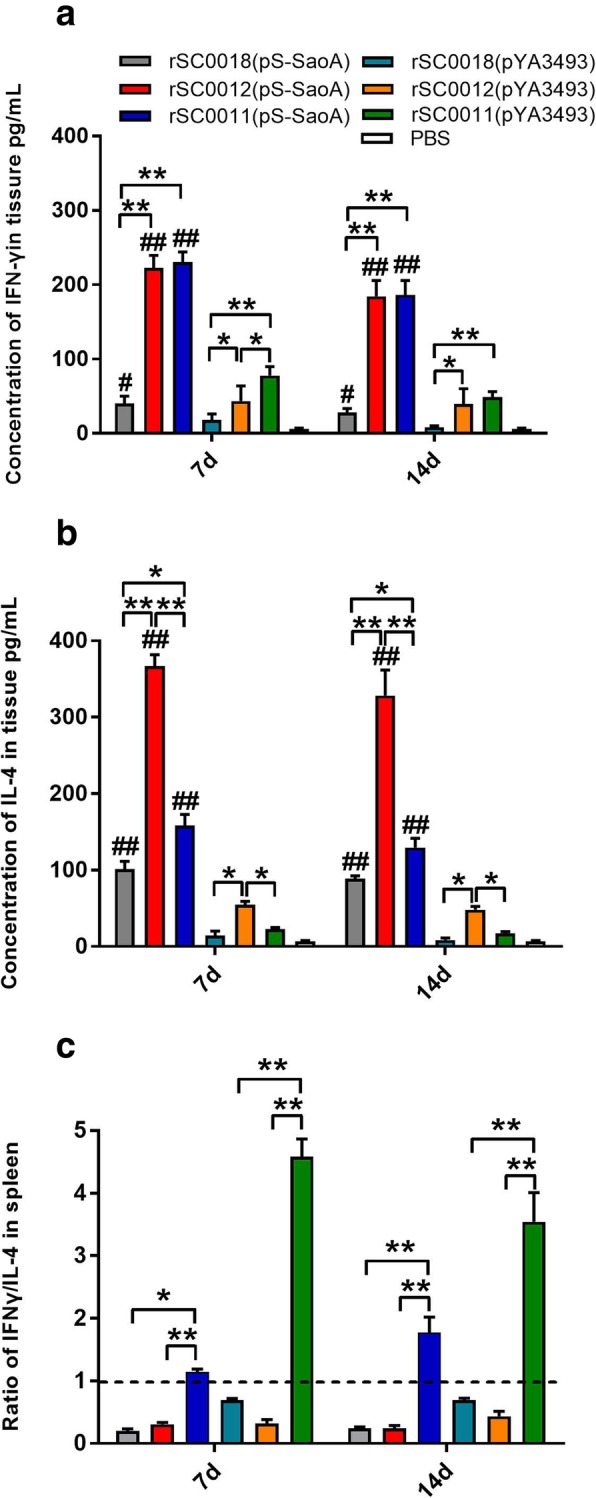
Cytokines levels in ten mice immunized with the S. Choleraesuis vaccines. IFN-γ (**a**) and IL-4 (**b**) in spleens 7 d after the booster dose were assayed with an EILSA kit. A PBS control were also included. (**c**) the ratio of IFN-γ to IL-4. The significant differences between groups of each strain was indicated. The assay was performed in duplicate and repeated at least 3 times. *, *P* < 0.05; ** *P* < 0.01, for strains rSC0011, rSC0012, rSC0018 with either pYA3493 or pS-SaoA compared each other; #, *P* < 0.05; ##, *P* <  0.01, for the strains rSC0011, rSC0012, rSC0018 with pS-SaoA compared to these strains with pYA3493 respectively at 7 d and 14 d post immunization

To further evaluate the effect of the ΔP_fur88_:: TT *araC* P_BAD_*fur* and ΔP_crp527_::TT *araC* P_BAD_*crp* mutations in a strain with multiple preexist mutations on Th1/Th2 immune responses, the levels of IFN-γ and IL-4 in the spleen tissues of mice 7 days and 14 days after booster immunization were measured. The results showed that the titers of IFN-γ and IL-4 induced in mice immunized with rSC0012, and rSC0011 with either pYA3493 or pS-SaoA were significantly higher than those induced in mice inoculated by strain rSC0018 with either pYA3493 or pS-SaoA at both 7 and 14 days after booster (Fig. 5a, b; *, *P <* 0.01,**, *P <* 0.05). Although rSC0011(pS-SaoA) induced a slightly higher level of IFN-γ in mice than rSC0012(pS-SaoA) did, no significant difference was observed in 3-week-old mice at both 7 and 14 days after booster (Fig. 5a-c). However, rSC0011(pYA3493) elicited a higher level of IFN-γ than rSC0012(pYA3493) did at 7 days after booster. The titers of IL-4 induced by rSC0012 with either pS-SaoA or pYA3493 in spleen tissues were significantly higher than those induced by rSC0011 with either pS-SaoA or pYA3493 in 3-week-old mice (Fig. 5b; *, *P* < 0.05, **, *P* < 0.01) at 7 and 14 days after booster. Of note, rSC0012(pS-SaoA) induced a dominant Th2 immune response (IFN-γ < IL-4, IFN/IL-4 < 1) in 3-week-old mice (Fig. 5c); whereas, rSC0011(pS-SaoA) elicited a moderately dominant Th1 immune response (IFN-γ > IL-4, IFN/IL-4 > 1) in 3-week-old mice (Fig. 5c, *, *P* < 0.05, **, *P <* 0.01). These results indicated the isogenic strain with two different mutations, ΔP_fur88_::TT *araC* P_BAD_*fur* or ΔP_crp527_::TT *araC* P_BAD_*crp*, affected different branches of immune responses. In additional, the titers of IFN-γ and IL-4 induced by the three attenuated strains harboring pS-SaoA in immunized mice were higher than those induced by the same strains harboring the emptor vector pYA3493 (Fig. 5a–c; #, *P* < 0.05, ##, *P* < 0.01), suggesting that the protein, SaoA, might augment the immune responses in mice.

### Induction of inflammation in mice

**Fig. 6:**
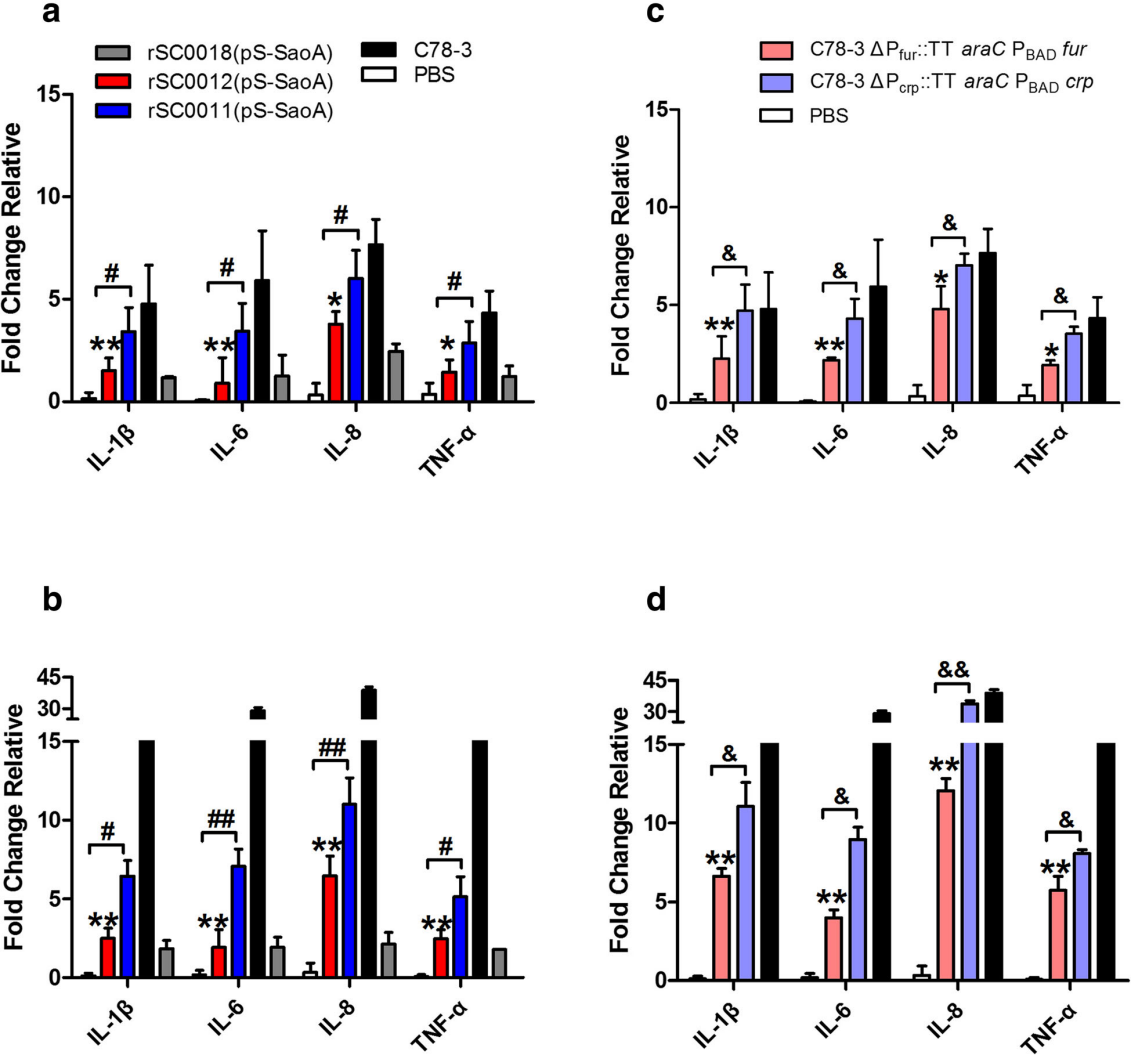
Induction of inflammatory cytokines at 6 h and 12 h post-infection in ten mice immunized with different *Salmonella* strains. Analysis of RNA transcript levels by qPCR showed that all mutant strains induced less inflammatory cytokine production than the wild-type strain C78-3 at both 6 h (**a** and **c**) and 12 h (**b** and **d**) post-infection. &, *P* < 0.05, C78-3 ΔP_fur88_ TT *araC* P_BAD_*fur* compared to C78-3; #, *P* < 0.05, ##, *P* < 0.01, rSC0012(pS-SaoA) compared to rSC0011(pS-SaoA); *, *P* <0.05, **, *P* < 0.01, strains with ΔP_fur88_ TT *araC* P_BAD_*fur* mutation compared to C78-3

The inflammatory properties of intestinal tissue were investigated in mice after immuned vaccine strains and wild type strain, C78–3.The expression of cytokine genes, TNFα, IL-1β, IL-6 and IL-8, were assessed by quantitative real -time PCR in gut tissue samples at 6 h and 12 h postinfection. All strains with mutation ΔP_fur88_::TT *araC* P_BAD_*fur* showed significantly lower transcription levels of cytokine genes IL-1β, IL-6, IL-8 and TNFα than the wild -type strain, C78–3 at both 6 h and 12 h postinfection (Fig. 6a-d,*, *P* < 0.05, **, *P <* 0.01). The C78–3 with mutation ΔP_fur88_::TT *araC* P_BAD_*fur* induced significantly lower transcription levels of cytokine genes IL-1β, IL-6,IL-8 and TNFα than the same strain with ΔP_crp527_::TT *araC* P_BAD_*crp* mutation at both 6 h and 12 h postinfection (Fig. 6c and d, &, *P* < 0.05, &&, *P <* 0.01). At 6 h postinfection, strain rSC0012(pS-SaoA) showed significantly lower transcription levels of cytokine genes IL-1β, IL-6, IL-8 and TNFα than strain rSC0011(pS-SaoA) (Fig. 6a), a similar trend was seen with IL-1β, IL-6, IL-8 and TNFα at 12 h, although the differences were not significant for IL-6 and TNFα (Fig. 6b,#, *P* < 0.05, ##, *P <* 0.01). These results suggest that the addition of the ΔP_fur88_::TT *araC* P_BAD_*fur* mutations could decreased the inflammatory potential of strains rSC0012(pS-SaoA).

### Comparison of the protective immunity induced by *S.* Choleraesuis strains

**Fig. 7:**
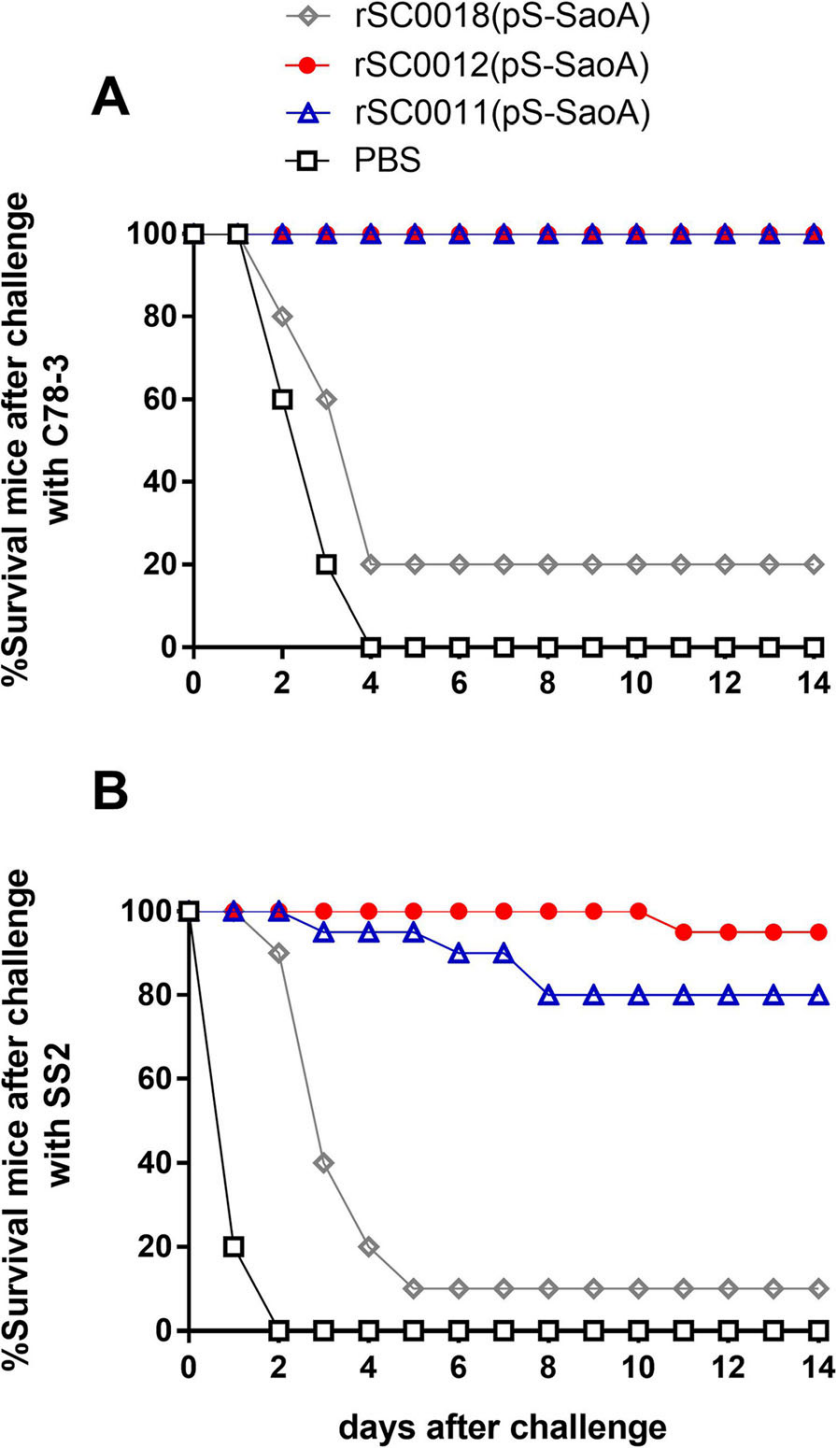
Protection in mice.Groups of 40 mice were orally immunized twice at 3‑weeks intervals with indicated strains. Half of the mice were challenged orally with 50×LD_50_ of C78-3 and the other half were intraperitoneally injected with 20×LD_50_ of *SS2* at 2 weeks after the 2nd immunization

To evaluate the protective immunity conferred by rSC0012(pS-SaoA), the mice in each immunized group were challenged orally with 50 × LD_50_ of the virulent *S*. Choleraesuis C78–3 strain or 20 × LD_50_ of the virulent SS2 strain at 14 days post-boost immunization. After challenge with C78–3, the results revealed 100% protection in mice immunized with either strain rSC0011(pS-SaoA) or strain rSC0012(pS-SaoA), suggesting full protection. Mice immunized with the rSC0018(pS-SaoA) strains resulted in 20% survival. In contrast, all the mice in the PBS group succumbed to the challenge after 4 days. There were no significant difference between the groups immunized with rSC0012(pS-SaoA) and rSC0011(pS-SaoA), though both displayed significantly higher levels of protection than the group immunized with rSC0018(pS-SaoA) (Fig. 7a). After the SS2 challenge, immunization with the rSC0011(pS-SaoA), rSC0012(pS-SaoA) and rSC0018(pS-SaoA) strains resulted in 80% survival, 95% survival and 16.7% survival with the lethal SS2 challenge, respectively. In contrast, all the mice in the PBS group succumbed to the challenge after 2 days.

## Discussion

The ultimate goal of an engineered live vaccine strain relies on achieving the proper balance between immunogenicity and attenuation [18, 19]. Achieving that goal will restrict unacceptable reactogenicity to avoid over-excitation of inflammatory responses, but sufficient metabolic activity should be maintained to enable the live vaccine to reach deep lymphatic tissues and induce protective immunity [20]. The development of bacterial vaccine relies on a combination of defined mutations [19, 21]. In order to enhancing the immunogenicity of vaccine strains or to disarm them, multiple independent defined mutations were introduced into *Salmonella* to generate new recombinant attenuated vaccine strains [6, 19, 22]. By coalescent proper mutations, vaccine strains can be befittingly designed to avoid unacceptable reactogenicity and enhanced immunogenicity [17]. Our previous live attenuated *S*. Choleraesuis vaccine vector rSC0011 occasionally caused enteritidis in mice [9]. One of the ways to address this problem is by incorporating a *sopB* mutation [5, 19]. In this paper, another way to improve the *S*. Choleraesuis vector was reported.

Fur is an important regulatory proteins of *Salmonella*, which has been implicated in the acid tolerance response since *fur* mutants are acid sensitive and cause altered expression of several acid shock proteins [10, 23]. A *S.* Typhimurium strain with an arabinose regulated *fur* mutation is adequately attenuated and highly immunogenic [6, 12]. However, *S*. Typhimurium belong to group B, while *S*. Choleraesuis group C. Whether the ΔP_fur88_::TT *araC* P_BAD_*fur* mutation that results in the proper balance between attenuation and immunogenicity of *S*. Typhimurium is also appropriate for attenuated *S*. Choleraesuis vaccine has not been reported previously.

We corroborated the *S*. Choleraesuis strain rSC0012 (ΔP_fur88_::TT *araC* P_BAD_*fur* Δ*pmi* Δ*relA*::*araC* P_BAD_*lacI* TT Δ*asdA*) displayed a regulated decrease of Fur production in the absence of arabinose. In a previous publication, *S*. Typhimurium strain with a single ΔP_fur88_::TT *araC* P_BAD_*fur* mutation has shown higher virulent than *S*. Typhimurium with a single ΔP_crp527_::TT *araC* P_BAD_*crp* mutation by an oral immunization. Unlike report from Curtiss et al. in *S*. Typhimurium [6], our studies did not showed that the ΔP_fur88_::TT *araC* P_BAD_*fur* mutation has higher virulent than ΔP_crp527_::TT *araC* P_BAD_*crp* mutation in *S*. Choleraesuis. In fact,the LD_50_ of rSC0012(pS-SaoA) was 38-fold higher than rSC0011(pS-SaoA) for 3-weeks-old mice with intraperitoneal injection. This result suggest that the ΔP_fur88_::TT *araC* P_BAD_*fur* mutation does different attenuation with *S*. Choleraesuis or *S*. Typhimurium,which may due to its complex role as a transcriptional activator of virulence in different strains [24].

The ΔP_fur88_::TT *araC* P_BAD_*fur* mutation can modify the iron regulated outer membrane protein, then strain with ΔP_fur88_::TT *araC* P_BAD_*fur* induced lower inflammation than the isogenic strain with ΔP_crp527_::TT *araC* P_BAD_*crp* mutation or wild type strain in mice. Theses results suggest that the *S*. Choleraesuis with ΔP_fur88_::TT *araC* P_BAD_*fur* mutation exhibits a lower tendency to trigger excessive inflammation while attenuated sufficiently.

Through the oral vaccination route, a vaccine strain will endure the challenges of acid, bile, and antimicrobial peptides existing in the gastrointestinal tract. Once inside Peyer’s patch, it will face macrophagocytes and T cells during the process transferring to deep lymphatic tissues [13]. Fur is essential to *Salmonella* for accessorial an adaptive acid tolerance response [10]. Although both rSC0011 and rSC0012 had similar levels of Peyer’s patch colonization by oral immunization,we observed that the rSC0012 was cleared more rapidly than the rSC0011 in deeper lymphatic tissues, spleen, and liver of mice. These results suggest that strain rSC0012 was more attenuated than rSC0011 in deep lymphoid tissue, which maybe due to an increase in acid sensitivity cause by loss of Fur makes the cell more susceptible to killing by macrophages.

Both rSC0012 and rSC0011 induced higher levels of IgA, IgG, IFN-γ, and IL4 responses with great colonization compared to strain rSC0018 with less colonization in mice. In general, the live *Salmonella* vaccines with RDAS display superior colonization level in lymphoid tissues during the invasion stage, leading to enhanced protection by effectively colonizing lymphoid tissues [6, 25]. However, there are do exist high levels of colonization but low immunogenicity. A strain with the regulated delayed *rfc* mutation exhibits superior colonization and yet does not stimulate higher heterologous protection than a *Δrfc* strain without RDAS [26]. Thus, in addition to colonization level, other determining factors may exist to induce the enhanced protection achieved by regulated delayed attenuation. From the above, Selecting the proper mutation is critical for vaccine development with RDAS. This study confirmed above statement. Although the colonization of rSC0012(pS-SaoA) in mice was less than that of rSC0011(pS-SaoA), rSC0012(pS-SaoA) stimulated stronger serum IgG and mucosal IgA responses than the rSC0011(pS-SaoA). This phenomenon suggests that strain with regulated delayed *fur* mutation may stimulate stronger antibody response with fewer bacteria than strain with regulated delayed *crp* mutation.

Both rSC0012 and rSC0011 aroused a Th1 cell-mediated response, as ostensived by the significant up-regulation of imprint Th1 cytokines, IFN-γ, the stimulator of Th1-type T cell immune response. It could partially be that the intracellular characteristics of *Salmonella enterica* cause them to be detected on the surface of APC through MHC-I molecules. This result is consistent with previous studies with *S*. Typhi [6]. Strain rSC0012 induced higher Th2 cell-mediated response than strain rSC0011, as aroused by the significant up-regulation of imprint Th2 cytokines, IL-4 and greater humoral responses than rSC0011 in mice. This phenomenon may be related to the secreting of heterologous antigen. The more heterologous antigens were secreted during infection, the more likely to trigger an IL-4-dominant response [27]. We found a larger secretion of the SaoA from rSC0012, which could be presented by MHC-II molecules. An additional factor may be due to an increase in acid sensitivity cause by loss of Fur makes the rSC0012 more susceptible to killing by macrophages, thus weakening rSC0012’s ability to survive in macrophages. The presence of *S*. *suis*-specific IgA serves to promptly deliver the antigen to Peyer’s patch dendritic cells or phagocytes and also promptly excite the adaptive immunity during secondary exposure [16]. Generally speaking, the more attenuated the vaccine strain is, the lower its immunogenicity is [17, 22], while, the more attenuated rSC0012 strain induced significantly higher IgA and IgG antibody responses to SaoA than the rSC0011 at 3 weeks after the initial immunization in juvenile mice. This may be the result of a equilibrium between attenuation and the ability to participation immune components and activate a controlled over-inflammatory response by the finely crafted strain [19].

The results presented herein highlight that strain rSC0012(pS-SaoA) with regulated delayed *fur* mutation has retained vaccine efficacy and adequate immunogenicity whilst being safer than previous strain rSC0011(pS-SaoA). Furthermore, the inclusion of the ΔP_fur88_::TT*araC*P_BAD_*fur* mutation may be able to decrease inflammation caused by live-attenuated *Salmonella enterica* serotype Choleraesuis vaccine, which has been an imperfection for other live-attenuated *Salmonella enterica* serotype Choleraesuis vaccine which transformed from wild type virulent strain. Both *S.* Choleraesuis and *S. suis* are major swine pathogens. Currently, there exist license live vaccines against *S. Choleraesuis* for swine, including Entersol® *Salmonella* T/C and SC-54 manufactured by Boehringer Ingelheim and Arugs SC/ST, respectively [28]. However, there is no licensed vaccine against *S. suis*. Preventing the diseases caused by *SS*2 in swine with a combined vaccine is a long-sought goal.

## Conclusions

Our results have shown the strains rSC0012(pS-SaoA) with regulated delayed *fur* mutation could confer higher protection against challenges with lethal doses of *SS*2 or *S*. Choleraesuis C78–3. Thus, the use of attenuated *S.* Choleraesuis to develop a vaccine against *S*. *suis* will have the great potential to ease the burden of both pathogens. The regulated delayed *fur* mutation in the novel vaccine rSC0012 resulted in a well-safety, highly immunogenic, and effective vaccine in mice, this study has paved the way for testing in piglets. Our findings will aid the optimal of a *S*. Choleraesuis vaccine vector capable of eliciting a suitable immune response against other pathogens.

## Methods

### Animals

Three-week-old female BALB/c mice were purchased from Animal Center of Yangzhou University, and kept 1 week before inoculation. All animal experiments were authorized by the Jiangsu Administrative Committee for Laboratory Animals (permission number SYXK-SU-2007-0005) and accorded to the Jiangsu Laboratory Animal Welfare and Ethics guidelines of the Jiangsu Administrative Committee of Laboratory Animals. All surgery was performed under anesthesia intraperitoneally injected with sodium pentobarbital, 40 mg per kilogram mouse weight. All the animals were humanely euthanized after the study by inhalation of CO_2_,while injection with sodium pentobarbital, 40 mg per kilogram mouse weight, and all efforts were made to minimize suffering.

### Strains, plasmids, and culture conditions

The strains, plasmids, used in this study are described in Table 2. C500, an approved live *S.* Choleraesuis vaccine strain attenuated by thallium compound in China, was used as an attenuation control [32]. The genetic characterization of this strain has been reported [29]. *S*. suis serotype 2 (SS2, CVCC3928) and *S*. Choleraesuis C78–3 (CVCC79103) were purchased from China Institute of Veterinary Drug Control. Plasmid pYA3493 is an Asd^+^ vector with a P_trc_ promoter. The *asd* gene from *Salmonella* was used as a unique plasmid marker to be used in *asd* mutants to constitute a balanced-lethal system [14]. LB medium [5],Nutrient broth (NB) and MacConkey agar (Difco) were used for phenotype characterization. When required, media were supplemented with 2,6-diaminopimelic acid (DAP;50 μg/mL),chloramphenicol (Cm; 25 μg/mL),L-arabinose (0.2% wt/vol), D-mannose (0.2% wt/vol) or sucrose (5% wt/vol). The empty plasmid vector pYA3493 and expression vector pS-SaoA were described on previous studies [5]. The *saoA* gene is under the control of the P_trc_ promoter (Table 2). *S.* Choleraesuis vaccine vector strain rSC0012 harboring plasmid pS-SaoA (expression vector) or pYA3493 (control vector) were grown in LB broth with both 0.2% arabinose and 0.2% mannose. Selenite broth was used for enrichment of *S.* Choleraesuis from mice tissues. Strains were prepared as previously described [5, 6, 9, 12]. Bacterial growth was monitored with a spectrophotometer at OD_600_ and by direct plating for colony counts.

**Table 2:** Strains and plasmids

Strain or plasmid	Relevant characteristics or genotype	Source or reference
*E. coli* strains		
BL21	F^−^*pT hsdSB* (rB^−^ mB^−^*al dcm* (DE3)	Invitrogen
χ7213	*thi-1 thr-1 leuB6 fhuA21 lacY1 glnV44 asdA4 recA1 RP4 2-Tc::Mu pir; Km*^*r*^	Gift from Dr. Curtiss, III
*Salmonella* Choleraesuis		
C78–3	Wild type, virulent, CVCC79103	Lab stock
C500	*S.* Choleraesuis vaccine strain attenuated by chemical mutation, CVCC79500	[29]
rSC0005	Δ*pmi-2426,* C78–3	[9]
rSC0008	Δ*pmi-2426* Δ*relA199::araC* P_BAD_*lacI* TT	This study
rSC0009	Δ*pmi-2426* Δ*relA199::araC* P_BAD_*lacI* TT ΔP_fur88_::TT *araC* P_BAD_*fur*	This study
rSC0011	ΔP_crp527_::TT *araC* P_BAD_*crp* Δ*pmi-2426* Δ*relA199::araC* P_BAD_*lacI* TT Δ*asdA33*	[9]
rSC0012	Δ*pmi-2426* Δ*relA199::araC* P_BAD_*lacI* TT ΔP_fur88_::TT *araC* P_BAD_*fur* Δ*asdA33*	This study
rSC0018	Δ*asdA33*	C500
	C78–3 ΔP_fur88_::TT *araC* P_BAD_*fur*	C78–3
	C78–3 ΔP_crp527_::TT *araC* P_BAD_*crp*	C78–3
*Streptococcus suis* serotype 2	Wild type, virulent, CVCC3928	Lab stock
Plasmids		
pYA3493	Asd^+^; pBR *ori*, P_trc_ promoter, β-lactamase signal sequence-based periplasmic secretion plasmid	[15]
pS-SaoA	pYA3493 with SaoA, P_trc_ promoter	[5]
pET28a	expression vector, T7 promoter; Km^r^	Novagen
Suicide vector		
pRE112	*sacB mobRP4 R6K oriV oriT Cm*^*r*^	[30]
pDMS197	*tet*^*r*^*sacB mobRP4 R6K oriV oriT*	[30]
pYA3832	ΔP_crp527_::TT *araC* P_BAD_*crp*, pRE112	[31]
pYA3546	Δ*pmi-2426*, pDMS197	[31]
pS003	Δ*relA199::araC* P_BAD_*lacI* TT, pRE112	[9]
pS005	ΔP_fur88_::TT *araC* P_BAD_*fur*	This study
pYA3736	Δ*asdA33*, pRE112	[9]

### Construction of *S.* Choleraesuis mutant strains

Four mutations ΔP_fur88_::TT *araC* P_BAD_*fur*, Δ*pmi*, Δ*relA*::*araC* P_BAD_*lacI* TT, and Δ*asdA* were introduced into *S.* Choleraesuis C78–3 by conjugation with *E. coli* χ7213 harboring aforementioned suicide vectors as previously described [33]. The suicide vectors used are listed in Table 2 and Fig. 1a. To construct mutation ΔP_fur88_::TT *araC* P_BAD_*fur,* a 1335-bp TT *araC* P_BAD_ cassette were used to replace the 239-bp promoter sequence of the *fur* gene to achieve arabinose-regulated Fur synthesis (Fig. 1a). The *araC* P_BAD_ cassette contains a transcription terminator (TT) sequence to prevent *araC* transcription reading through adjoining genes. Plasmid for ΔP_fur88_::TT *araC* P_BAD_*fur* were confirmed by DNA sequencing. All the primers used have been reported [21].

### Characterization of *S.* Choleraesuis mutations in vitro

All mutations were confirmed by colony PCR using homologous primers [12]. The Δ*asdA* mutation was verified by growth with or without DAP in LB broth [14, 15]. Lipopolysaccharide (LPS) profiles were examined by silver staining in 12% sodium dodecyl sulfate-polyacrylamide (SDS) gel for the Δ*pmi* mutation [33, 34]. The ΔP_fur88_::TT *araC* P_BAD_*fur* deletion-insertion mutation was verified by reduced production of Fur protein as arabinose concentrations decreased with the increased bacterial growth by western blot using anti-Fur antiserum [12]. The production of SaoA was verified by western blot using anti-SaoA antiserum, respectively [9,18,19,20,].

### *Salmonella* subcellular fractionation

To evaluate the subcellular localization of synthesized SaoA in the live attenuated *S.* Choleraesuis vaccine*,* cultures were grown in NB to an OD_600_ of 0.8 and centrifuged at 13,200×g for 5 min to collect supernatant and pellet. The culture supernatant was saved for later analysis of the bacterial secreted proteins. Periplasmic fractions were prepared using the lysozyme-osmotic shock method as previously described [27, 35]. Equal volumes of supernatant,periplasmic, cytoplasmic samples were separated by SDS-PAGE. Proteins were then transferred to polyvinylidene fluoride membrane for western blot analysis using anti-SaoA antiserum [5]. The gel band were analyzed with ImageJ software (NIH) [31].

### Preparation of SaoA and *Salmonella* outer membrane proteins (SOMPs)

His-tagged SaoA fusion protein and *Salmonella* outer membrane proteins (SOMPs) were prepared as previous studies [5].

### Determination of virulence in mice

Three-week-old female BALB/c mice were obtained from Animal Center of Yangzhou University. Mice were fasting for 6 h before inoculation. Recombination *S.* Choleraesuis vector strains were cultured in LB broth with D-0.2% mannose and 0.2% L-arabinose for 12 h at 37 °C as standing cultures. The cultures were diluted 1:50 in the same media and cultured at 37 °C to an OD_600_ of 0.9. Bacteria were collected by centrifugation at 13,200×g for 5 min at room temperature and resuspended in PBS to densities suitable for the inoculation. Serial dilutions of the *S.* Choleraesuis strains were plated onto LB agar supplemented with 0.2% D-mannose and 0.2% L-arabinose to measure the actual densities. Groups of five mice were inoculated with different doses in 20 μl (oral immunization) or 100 μl (intraperitoneal immunization). The mice were observed for 4 weeks for death. The LD50s were calculated according the method of Reed and Muench [10].

### Distribution of *Salmonella* in BALB/c mice

A colonization assay for recombination *S.* Choleraesuis vector strains was performed as described previously [9, 10]. Three-week-old female BALB/c mice were divided into 7 groups with 25 mice in each group. Each mouse was orally inoculated with 1 ± 0.2 × 10^9^ CFU of *S.* Choleraesuis strains. Peyer’s patches, spleen, and liver of the mice were collected on days 3, 7, 14, 21 and 28 post-inoculated. The densities of bacteria in the tissues were determined using the method reported in previous studies [5, 9, 10, 36]. The assay was performed twice, and the data were similar and pooled for analysis.

### Immunization of mice

Bacteria were grown and collected as above. Serial dilutions of the *S*. Choleraesuis vaccine strains were plated onto LB agar supplemented with 0.2% D-mannose and 0.2% L-arabinose to determine the actual dose. Three-week-old female BALB/c mice were orally inoculated with 1 ± 0.2 × 10^9^ CFU of *S.* Choleraesuis vaccine strains containing either pS-SaoA or pYA3493. Mice were boosted inoculated with the same dose of the same strain after 3 weeks. About 50 μL of whole blood was collected by tail vein 3 weeks after primary inoculation and 2 weeks after boosting. Serum was separated from the whole-blood samples and stored at − 70 °C. Vaginal-wash samples in mice were collected at the indicated time and stored at − 70 °C [9, 25, 30, 36]. This experiment was performed in triplicate with each group receiving a similar dose of the vaccine strains.

### Tissue collection after *Salmonella* infection of mice

Three-week-old female BALB/c mice were divided into 5 groups with 10 mice in each group. Groups of mice were orally inoculated with 1 ± 0.3 × 10^9^ CFU of *Salmonella* strains. Spleen and intestinum tenue of the mice were collected at 6 h and 12 h postinfection. The tissues were frozen with liquid nitrogen and then transferred to − 70 °C.

### Enzyme-linked immunosorbent assay (ELISA)

Serum IgG antibody production against *S.* Choleraesuis outer membrane proteins (OMPs) and SS2 SaoA,and vaginal-wash IgA antibody production against SaoA in mice were evaluated by Enzyme-linked immunosorbent assay (ELISA) [5]. Cytokines in tissues were analyzed by sandwich ELISA using commercial kits (BD Biosciences) according to manufacturer’s instructions. The results from the two experiments were pooled for statistical analysis.

### Quantitative real -time PCR (qPCR) for cytokines

For RNA isolation, gut tissues were homogenized and suspended in TRIzol® (Thermo Fisher Scientific,USA). Tubes were vortexed for 3 min to disrupt the tissues. Chloroform was added to TRIzol® -treated samples and the samples centrifuged at 13200×g for 10 min. The aqueous phase was separated out, and the RNA precipitated using precooled isopropanol. For quantitative real-time PCR, 1 μg of RNA was then reverse transcribed to cDNA. The primers were designed using Primer Blast (NCBI net) and synthesized by TSINGKE Biological Technology Co., Ltd. The sequences of the primers are listed in Table 3. Each sample were amplified using 7500 Fast Real -Time PCR Instrument (ABI,US) using Fast SYBR Green Master Mix (Thermo -Fisher Scientific). The results were using internal reference GAPDH as control for normalization, and the 2 ^^-ΔΔCt^ method was used to estimate the relative expression level of the mRNAs of target genes.

**Table 3:** Primer sequences

Gene		Sequence (5′-3′)
GAPDH	Forward	CTT AGC ACC CCT GGC CAA G
	Reverse	GAT GTT CTG GAG AGC CCC G
IL-1β	Forward	GTG TCT TTC CCG TGG ACC TT
	Reverse	AAT GGG AAC GTC ACA CAC CA
IL-6	Forward	GGC GGA TCG GAT GTT GTG AT
	Reverse	GGA CCC CAG ACA ATC GGT TG
TNF-α	Forward	ATG AGC ACA GAA AGC ATG A
	Reverse	AAG AGG CTG AGA CAT AGG C
IL-8	Forward	CTG CAA GAG ACT TCC ATC CAG
	Reverse	AGT GGT ATA GAC AGG TCT GTT GG

### Challenge with *S. suis* serotype 2 (*SS*2) and *S.* Choleraesuis (C78–3) in mice

Twenty mice in each group were challenged with *SS*2 by intraperitoneal (i.p.) injection with 2.4 × 10^8^ CFU of *SS*2 in 100 μl PBS at 5 weeks after primary immunization. The 50% lethal dose (LD_50_) of *SS*2 in BALB/c mice was 1.2 × 10^7^ CFU. Another twenty mice in each group were challenged orally with 4.8 × 10^4^ CFU of C78–3 in 20 μl PBS. The LD_50_ of C78–3 in 3-week-old BALB/c mice was 9.5 × 10^2^ CFU. Challenged mice were monitored for death daily for 15 days [9, 19, 21, 25].

### Statistical analysis

Statistical analyses on ELISA were presented as the geometric means and standard deviations for all assays. A Mann-Whitney U Test (GraphPad Software, Inc.) was applied to contrasting the distribution of the *S*. Choleraesuis in tissues of mice. The Kaplan-Meier method (SPSS Software) was used for obtain the survival fractions following i.p. challenge of immunized mice. A *P* value of 0.05 was considered statistically significant.

## Data Availability

All data generated or analysed during this study will be available from the corresponding author on reasonable request.

## Abbreviations

*S*.Choleraesuis: *Salmonella enterica* serotype Choleraesuis
*S. suis*: *Streptococcus suis*
Sao: Surface-anchored protein
SS2: *S.suis* serotype 2
RDAS: Regulated delayed attenuated strategies
LB: Luria broth
LD_50_: 50% lethal dose
APC: Antigen-Presenting Cells
MHCI: Major Histocompatibility ComplexI
Th1: T helper type 1
PBS: Phosphate buffer saline
LPS: Lipopolysaccharide
SDS: Sodium dodecyl sulfate-polyacrylamide
